# Case report: Diagnosis of *Talaromyces marneffei* infection in an HIV-negative patient with septic shock and high-titer anti-interferon gamma autoantibodies by metagenomic next-generation sequencing

**DOI:** 10.3389/fcimb.2023.1163846

**Published:** 2023-07-04

**Authors:** Rao Du, Yinhe Feng, Hui Mao

**Affiliations:** ^1^Department of Pulmonary and Critical Care Medicine, West China Hospital, Sichuan University, Chengdu, Sichuan; ^2^Department of Respiratory and Critical Care Medicine, People’s Hospital of Deyang City, Affiliated Hospital of Chengdu College of Medicine, Deyang, Sichuan, China

**Keywords:** *Talaromyces marneffei*, septic shock, HIV-negative patient, anti-interferon gamma autoantibodies, metagenomic next-generation sequencing

## Abstract

**Background:**

Sepsis is a life-threatening condition caused by a dysfunctional response to infection from the host. Septic shock, a subset of sepsis, caused by *Talaromyces marneffei* infection (talaromycosis) has rarely been reported. Owing to its slow culture and low yield, talaromycosis is typically misdiagnosed in HIV-negative patients as other infections, such as tuberculosis, bacterial pneumonia, and lung cancer, especially in non-endemic regions. Early and accurate diagnosis as well as efficient treatment options are required to improve prognosis.

**Method:**

A 30-year-old HIV-negative Chinese woman from a non-endemic area of *T. marneffei* was initially misdiagnosed with tuberculosis. She had a poor response to anti-tuberculosis treatment. On July 16, 2022, she was admitted to our hospital; the patient developed septic shock on the third day after hospitalization and was ultimately diagnosed with talaromycosis *via* metagenomic next-generation sequencing (mNGS).

**Result:**

The condition of the patient improved after appropriate treatment with amphotericin B. Furthermore, enzyme-linked immunosorbent assay results confirmed that the patient had a high-titer of anti-interferon gamma (IFN-γ) autoantibodies.

**Conclusion:**

HIV-negative individuals with anti-IFN-γ autoantibodies typically have relapsing, refractory, and fatal infections, such as talaromycosis, which is typically misdiagnosed in the initial course of the disease. This can lead to septic shock. Clinicians should be aware that they may encounter HIV-negative patients with *T. marneffei* infection in non-endemic areas. Thus, mNGS is an effective technology for detecting *T. marneffei* infection. Additionally, the detection of anti-IFN-γ autoantibodies in these patients would aid in knowing their susceptibility to fatal infections.

## 1 Introduction

Sepsis is a major cause of death in critical patients and affects more than 30 million people worldwide annually (Huang et al., 2019). *Talaromyces marneffei* is a dimorphic fungus endemic to Southeast Asia, including Thailand, Vietnam, and China (Hu et al., 2013), that can cause severe infection. Occurrence of *T. marneffei* infections have been predominantly reported in HIV-positive patients. However, an increasing number of cases have recently been reported in non-HIV-infected patients. Culturing *T. marneffei* is essential for its diagnosis. Nonetheless, owing to the critical condition of *T. marneffei* infections, long time required for culturing, and low yield, clinical demands cannot be met (Ning et al., 2018). Accurate pathogen identification is crucial for the early initiation of appropriate antimicrobial treatment and sepsis management (Cecconi et al., 2018a). Thus, new methods are required to identify pathogens quickly and accurately, particularly in patients with septic shock. Herein, we report the case of a 30-year-old HIV-negative Chinese woman presenting recurrent fever, cough, and chest and back pain, who was eventually diagnosed with talarmycosis. She experienced a septic shock and was ultimately diagnosed with talaromycosis *via* metagenomic next-generation sequencing (mNGS) at our hospital. Upon fluid resuscitation and timely antifungal therapy, her condition improved. Through enzyme-linked immunosorbent assay (ELISA), we confirmed that the patient had a high level of anti-interferon gamma (IFN-γ) autoantibodies.

## 2 Case description

**Figure 1:**
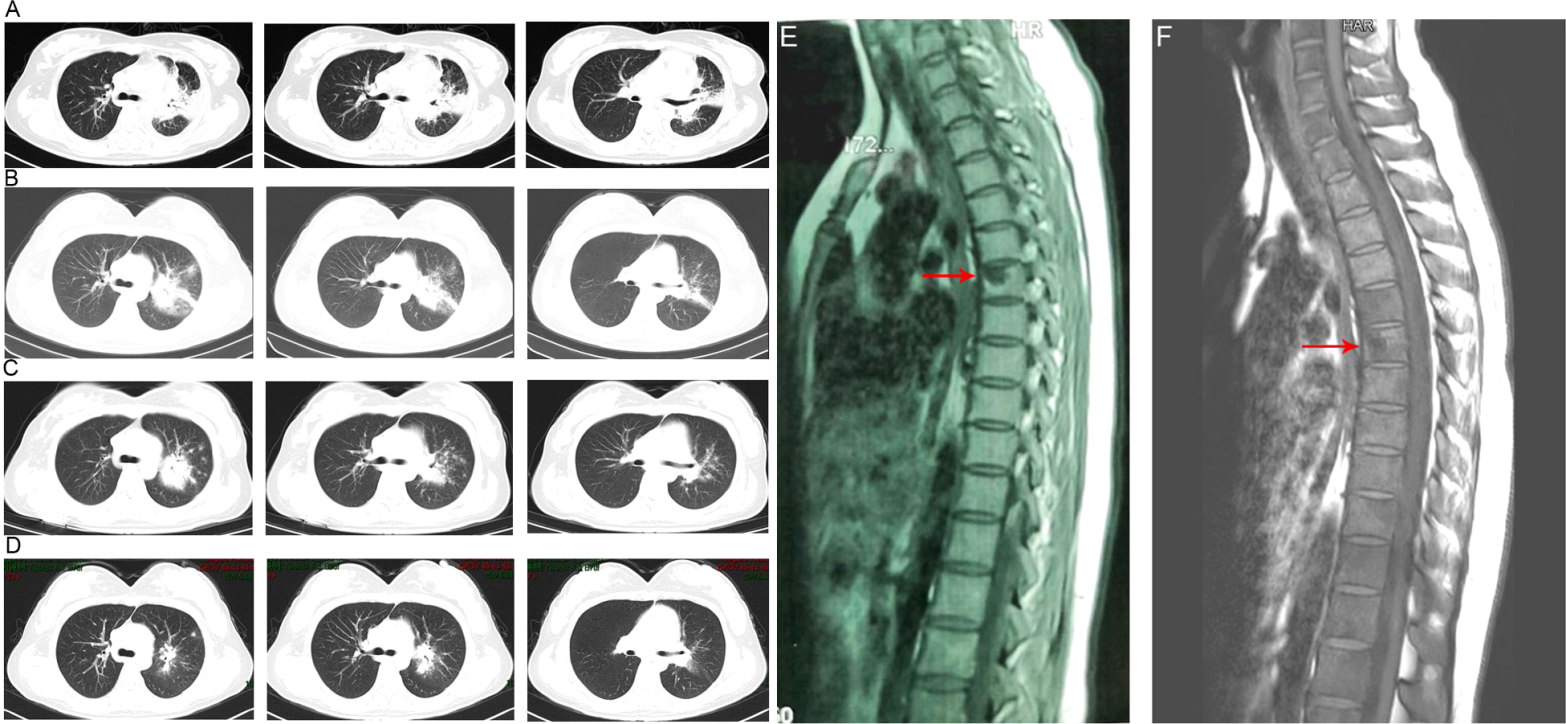
Chest computed tomography imaging results of the patient. **(A)** The chest CT before anti-tuberculosis treatment. **(B)** The chest CT before anti-*Talaromyces marneffei* treatment. Chest CT scans showed scattered patches, nodules, and ground-glass shadows in the left upper lobe with ill-defined borders and thickening of some interlobular septa. **(C)** The chest CT after 2 weeks of cumulative treatment with amphotericin B. The lesions in the left upper lobe of the lung were absorbed after two weeks of treatment with amphotericin B. **(D)** Chest CT scans of the local hospital after discharging from hospital 5 months. The lesions in the left upper lobe of the lung had been absorbed furthermore. **(E)**. The MRI of spine showed the lesions of thoracic vertebra. **(F)**. The MRI of spine showed the lesions of thoracic vertebra were absorbed after anti-fungal treatment.

**Figure 2:**
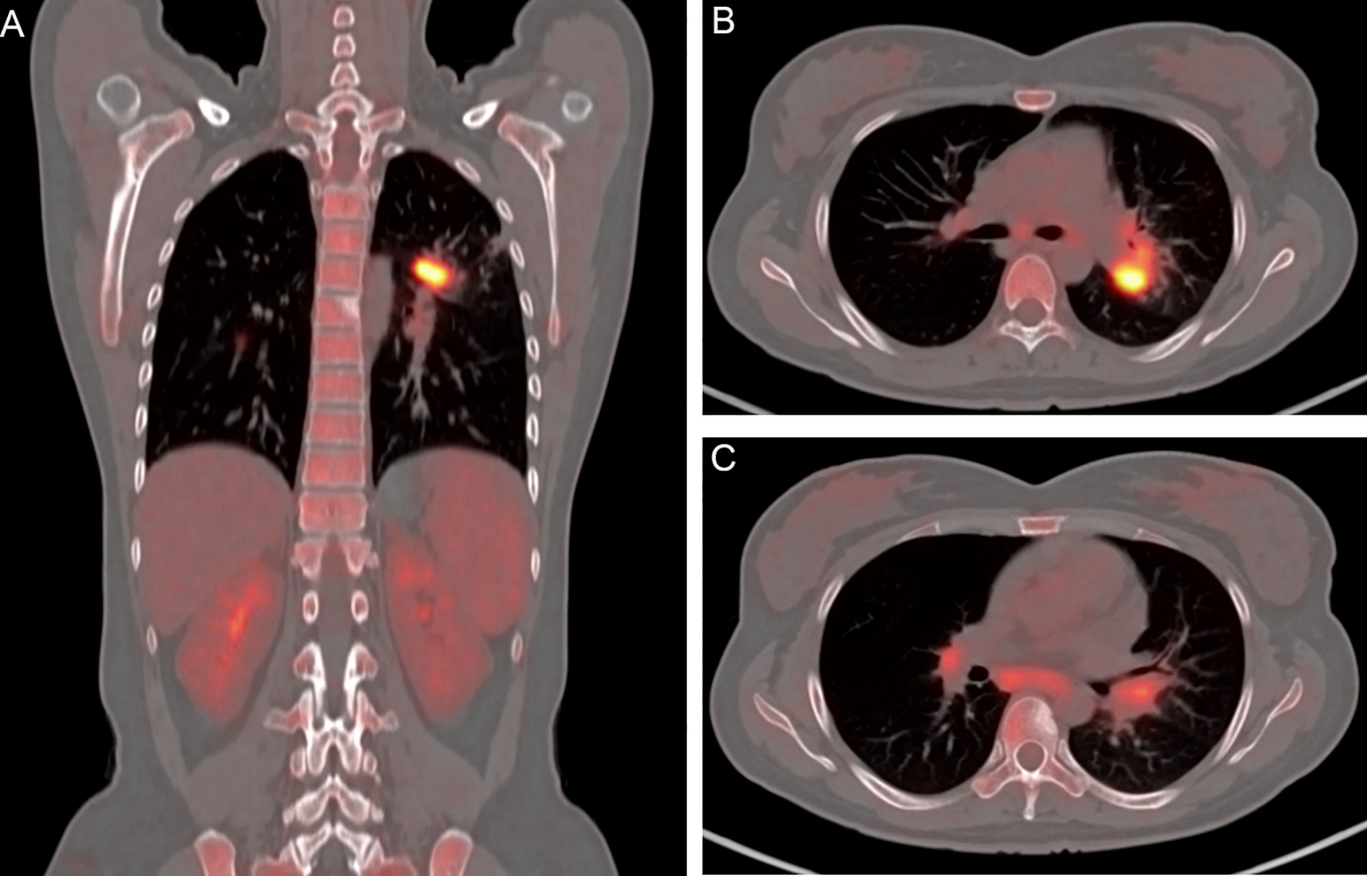
Positron emission tomography/X-ray computed tomography imaging results of the patient. **(A)** Thoracic (T-6) vertebral body showing low metabolism. **(B)** Left upper lobe of the lung showing high metabolism. **(C)** Osteolytic damage found in the vertebrae.

A 30-year-old Chinese female was admitted to our hospital with persistent fever and cough as well as and chest and back pain (lasting for 8 and 3 months, respectively). The maximum body temperature was 39.4°C, the fever was irregular. No other symptoms or signs of organ involvement was present. She had been initially admitted and treated at a local hospital. An examination at the local hospital showed an erythrocyte sedimentation rate of 59 mm/h. The HIV test yielded negative results; the results of the purified protein derivative test and interferon-γ release assay, which used the ELISA, were normal. The results of the acid-fast bacillus (AFB) test, smear, and cultures of the sputum and bronchoalveolar lavage fluid did not reflect any abnormalities. Even after 21-day antibiotic therapy (cefoperazone sulbactam), the patient showed no improvement. Considering her symptoms and the imaging features (**Figure 1A**) observed in her chest CT, she was prescribed anti-tuberculosis treatment comprising isoniazid, rifampicin, pyrazinamide, and ethambutol on December 15, 2021. She was non-responsive to the medications for up to six months. Meanwhile, her condition deteriorated, and she started experiencing chest and back pain. 2-deoxy-2-[fluorine-18] fluoro-D-glucose positron emission tomography/X-ray computed tomography (^18^F-FDG PET/CT) indicated that the thoracic (T-6) vertebral body showed low metabolism (**Figure 2A**), and the left upper lobe of the lung indicated high metabolism (**Figure 2B**). Chest CT scans showed some osteolytic damage in the vertebrae (**Figure 2C**). The patient was then transferred to our hospital for further diagnosis and treatment. The patient had no history of unprotected sex or blood transfusions. In addition, there was no history of other personal or hereditary diseases. She also denied traveling to any area except her hometown over the past 10 years. The vital signs during the initial examination were as follows: body temperature, 37.3°C; blood pressure, 107/74 mmHg; heart rate, 107 beats/min; and respiratory rate: 20 breaths/min. Physical examination was normal, except for tenderness at the 5/6 thoracic spine level. Initial laboratory examinations reported the following: white blood cell count, 13.83 × 10^9^/L; red blood cell count, 3.03 × 10^12^/L; hemoglobin level, 77 g/L; neutrophil percentage, 95.4%; lymphocyte percentage, 2.8%; and albumin level, 35.9 g/L. C-reactive protein (112.00 mg/L), procalcitonin (17.70 ng/mL), and interleukin-6 (26.40 pg/mL) levels and erythrocyte sedimentation rate (110.00 mm/h) showed significant elevation. Multiple peripheral blood cultures yielded normal results. The possibility of bacterial infection was first considered based on the clinical characteristics and auxiliary examinations. The physician intravenously administered piperacillin-sulbactam and levofloxacin to the patient. However, the patient was feverish, with a maximum temperature of approximately 39°C.

## 3 Diagnostic assessment

**Figure 3:**
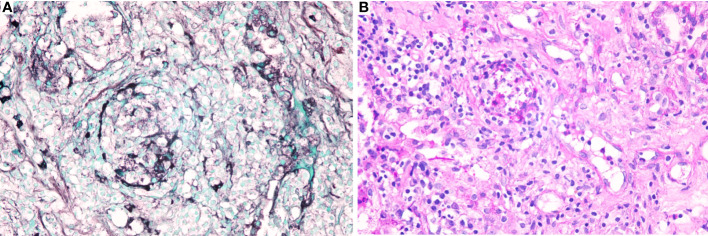
Specific stains of lung tissues. **(A)**. Hexamine silver staining of lung tissues was positive suspiciously; **(B)**. Periodic acid-Schiff (PAS) of lung tissues was positive suspiciously.

**Figure 4:**
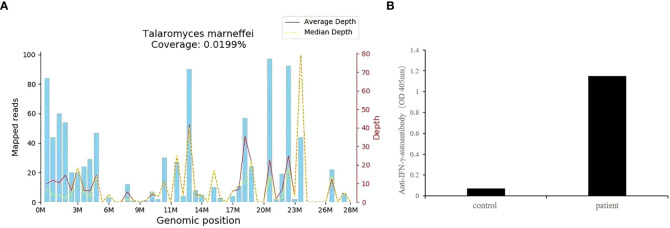
**(A)** *T. marneffei* coverage map. The identified reads were mapped to the *T. marneffei* reference genome (GenBank accession no. MN700106.1). The abscissa is the genome position of the *T. marneffei* reference genome. The left-side scale shows the number of matched mNGS sequences in the alignment. The right-side scale shows the sequencing depth (i.e., the number of times the base pair site has been sequenced). The blue bars show the number of matched mNGS sequences corresponding to various positions in the genome of the fungus. The red line represents the average sequence depth distribution at different positions in the fungal genome. The yellow line represents the median sequence depth distribution at different positions in the fungal genome. M, position in the genome in millions of base pairs (x-axis scale); **(B)** The OD of anti-IFN-γ-autoantibodies of this patient.

**Figure 5:**
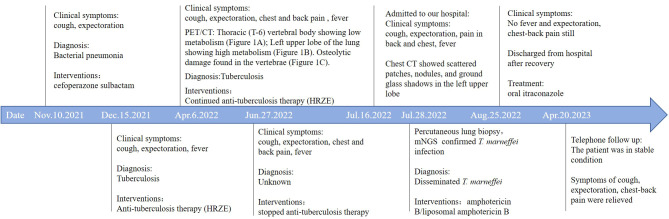
Timeline of the patient with relevant data on the episodes and interventions.

Owing to the poor response of the patient to antibiotic and anti-tuberculosis therapy, we further searched for potential evidence of any other kind of infection. The β-1,3-D-glucan test, *Aspergillus* galactomannan test, *Cryptococcus* antigen test, *Pneumocystis japonicum* DNA test, repeated purified protein derivative test, interferon-γ release assay (using the ELISPOT assay), and acid-fast staining and sputum culture were all negative for their associated infections. **Table 1** presents immunological reports of the patient under investigation. Chest CT scans showed scattered patches, nodules, and ground-glass shadows in the left upper lobe with ill-defined borders and thickening of some interlobular septa (**Figure 1B**). White blood cell count and C-reactive protein levels continuously increased, while hemoglobin and plasma albumin levels progressively decreased. Upon testing for them, we did not find any of the following antibodies in the patient: antinuclear antibodies, anti-double stranded DNA antibodies, anti-cyclic citrullinated peptide antibody, anti-keratin antibody, anti-neutrophil cytoplasmic antibodies, and HLA-B27. In conclusion, the diagnosis remained unclear. On the fifth day after admission to our hospital, the patient’s blood pressure decreased to 70/42 mmHg, and she developed asthenia. A large amount of fluid was administered for rehydration; and the blood pressure was not raised significantly with fluid resuscitation. Vasoactive drug (m-hydroxyamine 50mg) was used to raise blood pressure, her blood pressure then increased to 90/60 mmHg, and we adjusted her treatment to intravenous meropenem and levofloxacin. A percutaneous lung biopsy was performed after the patient’s condition had slightly improved, which showed granulomatous inflammation. Some pathogens were observed in the macrophages and interstitium suspiciously. Hexamine silver (**Figure 3A**) and periodic acid-Schiff (PAS) staining (**Figure 3B**) were positive suspiciously, whereas AFB, mucous card red staining, and TB-qPCR were negative. Tumors were not detected. Due to scarce availability of lung tissue and denial by the patient’s family, biopsy specimens were not sent for culturing. Eventually, we performed mNGS, which detected 1655 reads of *Talaromyces* spp. (Illumina NextSeq 550, Chengdu, China), including 1160 reads of *T. marneffei*, 435 reads of Human gamma herpesvirus 4 (EBV), no other bacteria and parasite had been detected. The MRI of spine showed the lesions of thoracic vertebra (**Figure 1E**). Thoracic cone biopsy was also performed to identify any thoracic vertebral lesions. mNGS of bone tissue revealed no pathogens. Pathological examination of the bone and bone marrow showed focal granuloma formation, and AFB smear, silver hexamine, PAS staining, and TB-qPCR results were negative. We could finally confirm *T. marneffei* infection *via* PCR amplification targeting the rDNA internal transcribed spacer region followed by a BLAST sequence comparison (https://blast.ncbi.nlm.nih.gov/Blast.cgi; GenBank accession no. MN700106.1, coverage 97%, identity 99.23%) (**Figure 4A**). These results showed that the patient was infected with *T. marneffei.* Anti-fungal therapy with amphotericin B was immediately initiated. She was treated with intravenous amphotericin B (0.6–1.0 mg/kg.d, ivgtt) as standard initial therapy. After 3 days of the antifungal treatment, the patient’s body temperature normalized, and the talarmycosis-associated clinical symptoms were relieved. ELISA confirmed that the patient had a high titer of anti-IFN-γ autoantibodies (1:2500), the value of optical density (OD) was 1.149, which was much higher than negative control (**Figure 4B**). During the antifungal treatment, the patient’s renal function was impaired. After discussing it with her family, we adjusted the antifungal therapy to liposomal amphotericin B (30 mg/d, ivgtt). Chest CT scans showed that the lesions in the left upper lobe of the lung had been absorbed after 2 weeks of cumulative treatment with amphotericin B (**Figure 1C**). The MRI of spine showed the lesions of thoracic vertebra were absorbed (**Figure 1F**). The main clinical diagnosis was severe *T. marneffei* pneumonia, spine *T. marneffei* infection, septic shock. Oral itraconazole was administered successively. Five months after discharge, there was no recurrence, which was confirmed *via* a telephonic follow-up, and Chest CT scans of the local hospital showed that the lesions in the left upper lobe of the lung had been absorbed furthermore (**Figure 1D**). A timeline of the patient with relevant data on the episodes and interventions is presented in **Figure 5**.

**Table 1:** Immunological details of the reported patient.

Laboratory investigations	Results		Normal range
	Initial results	Recovery phase	
Lymphocyte subset			
CD3^+^T (%)	58.46	71.46	66.90−83.10
CD4^+^T (%)	41.95	47.62	33.19−47.85
CD8^+^T (%)	15.23	22.00	20.40−34.70
CD4/CD8	2.75	2.16	0.97-2.31
CD3^+^T-cell (cell/µL)	105.00	2581	941.00−2226.00
CD4^+^T-cell (cell/µL)	75.00	1720	471.00−1220.00
CD8^+^T-cell (cell/µL)	27.00	795	303.00−1003.00
B-cell (%)	14.26	13.18	3.91−8.59
NK-cell (%)	24.04	14.22	9.26−23.92
B-cell (cells/µL)	26.00	476	175.00−332.00
NK-cell (cells/µL)	43.00	514	154.00−768.00
Immunoglobulin profile			
IgG (g/L)	21.90	16.9	8.00−15.50
IgA (mg/L)	4030.00	4800	836−2900
IgM (mg/L)	691.00	933	700.00−2200.00
IgE (IU/mL)	184.00	NA	5.00−150.00

NA, not available.

## 4 Discussion

Sepsis is a dysregulated host response to infection and is associated with acute organ dysfunction. Its incidence is high, and it remains a leading cause of death globally (Cecconi et al., 2018a). Depending on the patient’s underlying circulatory, cellular, and metabolic abnormalities, the risk of mortality from septic shock can substantially increase (Cecconi et al., 2018a). The most common site of infection that can lead to sepsis in the intensive care unit is the lung (Vincent et al., 2009). In addition to gram-positive and gram-negative bacteria, fungal organisms can also lead to sepsis, and its incidences are rapidly increasing recently (Martin, 2012). Early initiation of appropriate antimicrobial therapy and restoration of tissue perfusion *via* fluid resuscitation are important for the initial management of sepsis.

*T. marneffei*, previously named *Penicillium marneffei*, is a dimorphic fungus that exhibits mycelial and yeast forms at 25 and 37°C, respectively (Andrianopoulos, 2020). In China, it is primarily distributed in southern regions such as Guangdong and Guangxi (Hu et al., 2013). The first case of infection in humans was reported in 1973 (DiSalvo et al., 1973). However, the mechanism through which *T. marneffei* infects humans remains unknown. Reportedly, some subspecies of bamboo rats are mammalian reservoirs of this pathogen; nonetheless, the source of human infection is unclear (Deng et al., 1988). A key risk factor is contact with the endemic region of *T. marneffei* (Zeng et al., 2015). The longest incubation period is 10 years; however, most patients present their first symptoms in 6–12 months after leaving the endemic areas (Zheng et al., 2015). Nevertheless, our patient was from Guizhou and resided there all her life. She denied traveling to any talaromycosis-endemic area in the past 10 years. Therefore, we could not trace the site of infection in her case.

*T. marneffei* infection is typically found in patients with acquired immunodeficiency syndrome (AIDS), and it is the third most common opportunistic infection following tuberculosis and cryptococcosis in patients with AIDS, in tropical Southeast Asia (Vanittanakom et al., 2006). However, an increasing number of cases have been reported in HIV-negative patients with primary or secondary immunodeficiency, such as inborn errors of immunity (IEIs), autoimmune diseases, cancer, undergoing immunosuppressive therapy, and anti-IFN-γ autoantibody syndrome (Chan et al., 2016). IEIs includes STAT1 gain-of-function, IL-2 receptor common gamma chain deficiency, adenosine deaminase deficiency, CD40 ligand deficiency, and STAT3 deficiency, CARD9 deficiency, IFN-γ receptor 1 deficiency, RelB deficiency, and NFKB2 deficiency (Liu et al., 2022; Wang et al., 2022).

Anti-IFN-γ autoantibody (AIGA) syndrome is an emerging adult-onset immunodeficiency syndrome that was first described in 2004, characterized by high anti-IFN-γ autoantibody levels (Höflich et al., 2004). This association was first described in eight Chinese patients (Chan et al., 2016) and is primarily related to genetic variation (Guo et al., 2020). HLA class II alleles DRB1*16:02–DQB1*05:02 and HLA-DRB1*15:02–DQB1*05:01 are strongly associated with it (Guo et al., 2020). These patients are susceptible to infection by intracellular pathogens including *T. marneffei*, non-tuberculosis mycobacteria, and *Cryptococcus neoformans*. This syndrome has a considerably high mortality rate of 32%, and patients die at a median time of 25 months after diagnosis (Wipasa et al., 2018). AIGA-positive patients have a predisposition to the occurrence of multiple opportunistic infections (Shih et al., 2021) and disseminated infections and poor prognosis after standard antifungal treatment (Chen et al., 2021). Studies have revealed that 20.41% of HIV-negative adult patients with *T. marneffei* infection was AIGA-positive (Qiu et al., 2021). Compared to AIGA-negative patients, AIGA-positive patients with *T. marneffei* infection have fewer complications with underlying respiratory diseases but are more likely to be associated with the bone (Chen et al., 2021). Anti-IFN-γ autoantibodies significantly suppress the CD4^+^ Th1-cell immune response, leading to the failure of pathogen clearance by the host. Even with a normal lymphocyte count, the activation and proliferation of CD4^+^ T cells are impaired (Hu et al., 2021). Similarly, patients with autoantibody titers >2000 ng/mL showed strong inhibition of their CD8^+^ T cells (Chen et al., 2022). The titer significantly increases as the condition worsens during the disease course (Qiu et al., 2022). In our case, the titer of IFN-γ autoantibodies was 2500 ng/mL, and the counts of CD4 T and CD8 T cells were 75 and 27 cells/µL, respectively, which are considerably lower than the normal range, indicating strong inhibition of CD8^+^ T cells.

According to the immunoglobulin profile, the patient’s IgG, IgA, IgE increased, while IgM decreased slightly, which was consistent with the study of Chen et al. (Chen et al., 2020). From the literature review, we can see that the immunoglobulin profile was not the same, some patients showed high IgM, some showed low IgM (You et al., 2021; Li et al., 2023). The reported trend of IgA, IgE and IgM in HIV-Negative patients with disseminated *Talaromyces marneffei* infection with high-titer anti-interferon gamma autoantibodies is not completely consistent, indicating that patients are heterogeneous. Keragala et al. guessed the initial low CD4, CD3, CD8 counts would have been due ongoing sepsis and after the infection is controlled the counts improved (Keragala et al., 2020). On the recovery stage, the blood routine data showed the neutrophil percentage, 53.6%; lymphocyte percentage, 36.0%. NK cells, B cells, CD3, CD4 and CD8 T cells were 514 cell/µL, 476 cell/µL, 2581 cell/µL, 1720 cell/µL, and 795 cell/µL respectively. The IgG was 16.90g/L, IgM and IgA was 933.00mg/L,4800.0mg/L respectively (**Table 1****)**. Based on those, we suspected the changes of the immune function was secondary to the severe infection or due to the anti- IFN-γ-autoantibodies. The immune deficiency mechanism of anti-IFN-γ autoantibodies may include inhibition of the CD4 + T cells’ IFN-γ/pSTAT-1/Th1 pathway, ultimately leading to a severely compromised Th1 response. (Qiu et al., 2022). Regrettably, due to the refuse of patients’ family, the patient didn’t do genetic testing, we cannot know if the patient had STAT1 gain-of-function (GOF) mutations.

The clinical manifestations of *T. marneffei* infection are nonspecific and diverse and can present with fever, cough, expectoration, weight loss, skin lesions, generalized lymphadenopathy, and hepatomegaly (Zhou et al., 2014), which are generally atypical and may be confounded by the manifestations of other infectious diseases, such as tuberculosis. Both can cause fever, cough, expectoration, and bone damage. Owing to the smear staining microscopy, low positive rates of clinical samples, and long culture times, no single method can conclusively diagnose all tuberculosis cases (Jain et al., 2020). Thus, clinical examination, imaging, AFB smear, tuberculosis culture, molecular methods, histologic findings, and therapeutic approaches are needed. Purified protein derivative tests and TB-interferon-γ release assays can lead to the identification of false-negative patients with insufficient numbers or poor T-cell function (Diel et al., 2012). In our patient, the CD4^+^ T cells count was 75 cells/µL, and she was immunodeficient. That could be one reason why she was infected with *M. tuberculosis* and/or *T. marneffei*. Considering the chest CT manifestations and bone destruction, tuberculosis could not be ruled out as there was no evidence of other pathogens. However, the symptoms did not resolve after 6 months of anti-tuberculosis treatment, indicating the need for improved pathogen diagnostic technologies.

The diagnosis of *T. marneffei* has traditionally been confirmed by isolating the fungus from clinical specimens. Owing to the prolonged incubation time, prompt diagnosis is challenging. Although several antigen and antibody detection assays have recently been developed, they do not meet clinical demands (Wong et al., 2001). mNGS is a newly developed nucleic acid detection technology that can assist in the detection of infectious pathogens, particularly fastidious bacteria, slow-growing bacteria, and pathogens that cannot be isolated and cultured (Gu et al., 2019). The target-free detection strategy makes relying on any other prior test results unnecessary, which is more advantageous than broad-spectrum PCR. In terms of the time required to obtain results, using mNGS is considerably faster than culturing. For pathogens that cannot be cultured routinely, mNGS can quickly detect them in samples within 30 h (Zuo et al., 2023). In our case, the results of the routine pathogen detections were negative. When the pathologist could not diagnose the pathogen by microscopic morphology alone, the physician obtained a positive result for the presence of *T. marneffei* within 48 h using mNGS. After treatment with amphotericin B and amphotericin B liposomes, the patient’s lung and thoracic cone lesions diminished, and she was discharged. This reflects the advantages of mNGS in the detection of rare pathogens.

Amphotericin B is the first-line treatment for talaromycosis-affected patients; however, itraconazole and voriconazole are also effective (He et al., 2021). Considering the patient’s economic conditions, oral itraconazole (cheaper than voriconazole) was administered successively after 2 weeks of cumulative treatment with amphotericin B. The results were satisfactory.

In conclusion, for patients with recurrent fever, lung mass, and bone damage, in addition to tuberculosis and tumors, the possibility of *T. marneffei* infection and other rare pathogenic infections should be considered. mNGS could be an effective technology for *T. marneffei* detection in cases with septic shock. This case report aims to raise the awareness of physicians regarding the possibility of talaromycosis in non-endemic areas and HIV-negative patients. Anti-IFN-γ autoantibodies should be tested, particularly in patients with curtained recurrent infections like talaromycosis. Awareness of this disease among HIV-negative patients may promote prompt diagnosis, timely treatment, and better health outcomes.

## Data availability statement

The data presented in the study are deposited in the online repository, the names of the repository/repositories and accession number(s) can be found below: https://www.ncbi.nlm.nih.gov/ and PRJNA987339.

## Ethics statement

Ethical review and approval was not required for the study on human participants in accordance with the local legislation and institutional requirements. The patients/participants provided their written informed consent to participate in this study. Written informed consent was obtained from the individual(s) for the publication of any potentially identifiable images or data included in this article.

## Author contributions

RD and YF collected and interpreted the data. RD drafted the manuscript. HM revised and edited the manuscript. All authors contributed to the article and approved the submitted version.
